# Successful Partnerships: Exploring the Potential of Immunogenic Signals Triggered by TMZ, CX-4945, and Combined Treatment in GL261 Glioblastoma Cells

**DOI:** 10.3390/ijms22073453

**Published:** 2021-03-26

**Authors:** Lucía Villamañan, Laura Martínez-Escardó, Carles Arús, Victor J. Yuste, Ana P. Candiota

**Affiliations:** 1 Unitat de Bioquímica de Biociències, Departament de Bioquímica i Biologia Molecular, Universitat Autònoma de Barcelona, 08193 Cerdanyola del Vallès, Spain; lucia_1303@hotmail.es (L.V.); Carles.Arus@uab.cat (C.A.); 2 Cell Death, Senescence and Survival Group, Department of Biochemistry and Molecular Biology and Institute of Neurosciences, Faculty of Medicine, Universitat Autònoma de Barcelona, 08193 Cerdanyola del Vallès, Spain; lauramescardo@gmail.com (L.M.-E.); Victor.Yuste@uab.cat (V.J.Y.); 3 Centro de Investigación Biomédica en Red Sobre Enfermedades Neurodegenerativas (C.I.B.E.R.N.E.D.), Universitat Autònoma de Barcelona, 08193 Cerdanyola del Vallès, Spain; 4 Centro de Investigación Biomédica en Red en Bioingeniería, Biomateriales y Nanomedicina (CIBER-BBN), 08193 Cerdanyola del Vallès, Spain; 5 Institut de Biotecnologia i de Biomedicina (IBB), Universitat Autònoma de Barcelona, 08193 Cerdanyola del Vallès, Spain

**Keywords:** preclinical glioblastoma, cancer immune cycle, immunogenic signals, calreticulin, ATP, protein kinase CK2, temozolomide

## Abstract

Background: The relevance of the cancer immune cycle in therapy response implies that successful treatment may trigger the exposure or the release of immunogenic signals. Previous results with the preclinical GL261 glioblastoma (GB) showed that combination treatment of temozolomide (TMZ) + CX-4945 (protein kinase CK2 inhibitor) outperformed single treatments, provided an immune-friendly schedule was followed. Our purpose was to study possible immunogenic signals released in vitro by GB cells. Methods: GL261 GB cells were treated with TMZ and CX-4945 at different concentrations (25 µM–4 mM) and time frames (12–72 h). Cell viability was measured with Trypan Blue and propidium iodide. Calreticulin exposure was assessed with immunofluorescence, and ATP release was measured with bioluminescence. Results: TMZ showed cytostatic rather than cytotoxic effects, while CX-4945 showed remarkable cytotoxic effects already at low concentrations. Calreticulin exposure after 24 h was detected with TMZ treatment, as well as TMZ/CX-4945 low concentration combined treatment. ATP release was significantly higher with CX-4945, especially at high concentrations, as well as with TMZ/CX-4945. Conclusions: combined treatment may produce the simultaneous release of two potent immunogenic signals, which can explain the outperformance over single treatments in vivo. A word of caution may be raised since in vitro conditions are not able to mimic pharmacokinetics observed in vivo fully.

## 1. Introduction

Glioblastoma (GB) is the most prevalent malignant primary brain tumor in adults, and even after aggressive treatment, its prognosis is poor [1,2], leaving much room for improvement. The current standard chemotherapeutic treatment for GB in clinical practice is the alkylating agent temozolomide (TMZ), which is described as causing DNA damage through the production of O6-methyl adducts in guanine residues [3]. TMZ was historically thought to display a damaging effect on GB tumors through massive cell death due to DNA lesions and concomitant cell cycle arrest [4,5]. However, more recent work indicates that the immune system may also be involved in GB response to TMZ [6,7,8,9].

**Figure 1:**
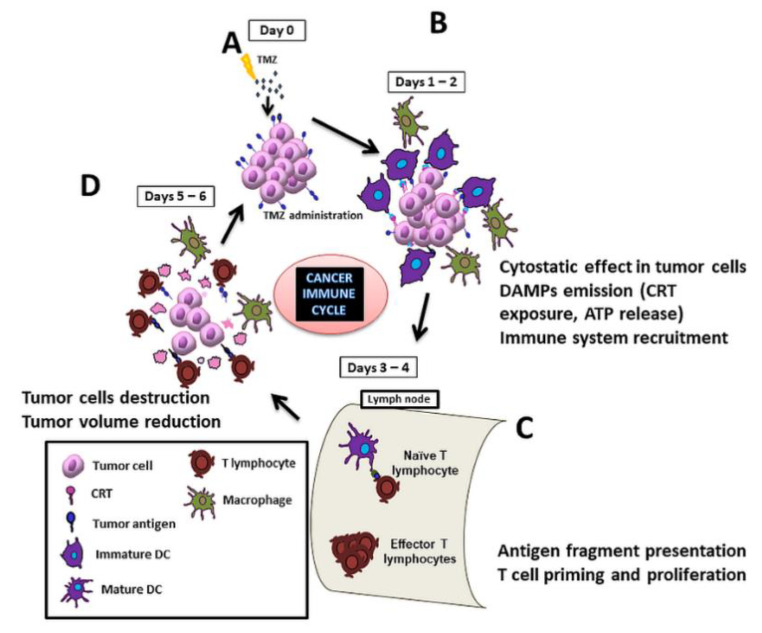
Scheme of the cycle for immune response elicitation against a preclinical glioblastoma (GB) during a full therapy cycle. When treated with temozolomide (TMZ) (**A**), tumor cells release/expose immunogenic signals, such as calreticulin (CRT), which attract dendritic cells (DCs) and macrophages (**B**). DCs migrate to proximal lymph nodes and prime naïve T-cells, which start their proliferation [19]. TMZ (or any antiproliferative agent) should not be administered during this period since it would interfere with lymphocytes’ proliferation and hamper proper immune response (**C**). At days 5–6 of the cycle, effector T cells reach the lesion site and attack tumor cells (**D**).

Immunotherapy is one of the most investigated new therapeutic approaches for treating cancer. It mostly works by either triggering the activation of the immune system or avoiding its inactivation [10,11]. One modality of immunotherapy actually profits from the use of certain drugs, called immunogenic, which are able to induce an antitumoral immune response by the elicitation of immunogenic cell death (ICD) [12]. This ICD is a non-tolerogenic form of cell death triggering an effective immune response against tumor cells through the activation of both the innate and adaptive host immune systems. Immunogenic drugs cause tumor cells to expose and/or release molecules called damage-associated molecular patterns (DAMPs), which may interact with immune system elements, triggering an effective antitumor response. Two of the most accepted DAMPs described in the literature are calreticulin (CRT) exposure and ATP release. CRT exposure is one of the first events observed in the ICD cascade, being a potent “eat-me” signal, facilitating the engulfment of tumor cells by antigen-presenting cells (APCs) and consequently leading to tumor antigen presentation and specific cytotoxic T lymphocyte (CTL) responses [13,14]. Some studies also suggest that CRT could be taken up by macrophages, inducing M1-polarization [15], also being relevant in the general process of removing damaged cells [16]. ATP release is a well-known “find-me” signal released from apoptotic/damaged cells, implicated in the activation of the NLRP3 inflammasome and contributing to the CD8+ T cell polarization and dendritic cells (DCs) maturation [17,18]. See Figure 1 for a schematic representation of the cancer immune cycle after an immunogenic drug hit.

Still, regarding cancer treatment, small molecular weight inhibitors of protein kinases have been a popular tool amongst targeted experimental therapies for GB [20,21]. Among them, inhibitors of protein kinase CK2 have been proven to be successful in preclinical studies for cancer treatment, including GB [22,23]. CK2 is a pleiotropic serine–threonine kinase implicated in a multitude of prosurvival and anti-apoptotic pathways [24]. Its overexpression contributes to creating an anti-apoptotic environment; CK2 activity downregulation has been described as producing tumor cell destruction [25,26]. Several molecules have been described as CK2 inhibitors, among them, CX-4945 which is an ATP binding site competitive inhibitor, which has been evaluated in several clinical trials (https://clinicaltrials.gov/ct2/home, (accessed on 25 March 2021) NCT01199718, NCT02128282, NCT00891280).

The use of animal models in oncological research is a key step in the search for new therapeutic strategies. GL261 cells grown in C57BL/6 mice is one of the most investigated immunocompetent models, widely used in GB preclinical studies [27,28,29,30,31]. Wu and Waxman [32] described an immune friendly cyclophosphamide treatment triggering activation of the immune system against GL261 subcutaneous tumors, which also proved useful with TMZ treatment of an orthotopic GL261 GB model, improving mice survival [29,33] in comparison with previous protocols [34]. Our group has coined the expression Immune Enhanced metronomic Schedule (IMS) to refer to such an “immune friendly” administration protocol [35].

We have been working towards the improvement in preclinical GB outcome and non-invasive therapy response assessment, with special interest not only in TMZ but also in its combination with another non-mutagenic agent, such as CX-4945. Our previous study showed that the combined TMZ plus CX-4945 treatment, also in an IMS schedule, demonstrated improved survival rates in comparison with single IMS TMZ treatment [29] (54.7 ± 11.9 days in the combined treatment vs. 38.7 ± 2.7 days in single TMZ treatment). The IMS administration of CX-4945 itself only produced a slight, although significant, survival improvement (untreated: 22.5 ± 1.2 days and CX-4945: 24.5 ± 2 days [29]), which was not observed in continuous or alternate days treatment schedule.

Overall, our previous results suggested that synergism between TMZ and CX-4945 actions was taking place, provided the IMS protocol was followed, producing better results than each agent alone. Based on those results, we decided to go back to in vitro experiments to unravel the mechanistic rationale for results obtained in vivo. We hypothesized, based on previous data described by Wu et al. [36] obtained with subcutaneously injected GL261 cells into C57BL/6J mice treated with cyclophosphamide, that a sustained antitumor immune response may be behind the survival increase when an IMS schedule using TMZ and CX-4945 is followed [29], since therapeutic antiproliferative administration is avoided during the amplification step shown in Figure 1. To further evaluate this possibility, we proceeded to study whether these drugs would produce additive/complementary immunogenic cell death, able to elicit the host immune system better, and, ultimately, improve the survival of treated mice. To assess this possibility, in vitro studies were carried out, and release/exposure of DAMPs was evaluated in GL261 cultured cells, as well as their potential for inducing cell death in tumor cells.

Thus, the main goal of this work was to gain insight into TMZ and CX-4945 treatment, either single or combined treatment in GL261 cultured cells, having in mind the satisfactory in vivo results obtained with GL261 GB tumor-bearing mice. The characterization of their in vitro cytotoxic/cytostatic effect and immunogenic cell death signals profile should help to understand and explain the rationale of the combined treatment opening the door to future optimization. To the best of the author’s knowledge, this is the first study assessing the immunogenic potential of CX-4945 in GL261 GB cells.

## 2. Materials and Methods

### 2.1. GL261 Cell Culture

GL261 murine glioma cells were obtained from the Tumor Bank Repository at the National Cancer Institute (Frederick, MD, USA) and cultured as previously described by our group in [37]. Mouse short tandem repeat (STR) profile and interspecies contamination were assessed, as well as PCR evaluation to discard Mycoplasma and virus contamination.

### 2.2. Cell Treatments

GL261 cells were treated with temozolomide (TMZ, T2577, Sigma–Aldrich, Darmstadt, Germany), CX-4945 (S2248, Selleckchem, Houston, TX, USA), and doxorubicin (Doxo 44583, Sigma–Aldrich, Darmstadt, Germany). Different concentrations and time point treatments were studied depending on the question being addressed (See Table 1 for ranges). GL261 cells (3500–500,000, depending on the test) were seeded in multiwell plates (6, 24, or 96 wells) or 60 mm dishes both from Sarstedt, and treatment was maintained during 12–72 h.

### 2.3. Cell Viability Assays

#### 2.3.1. Trypan Blue Exclusion Assay

GL261 cells were seeded in a 24-well plate at a seeding density of 32,000 cells per well. After 24 h, the medium was removed, and new medium with the desired treatment was added. TMZ (0.5–4 mM) and CX-4945 (25–100 µM) treated cells were analyzed. After the treatment period (24–72 h), the medium was collected, cells washed with phosphate buffered saline (PBS), and trypsinized (0.5 mL of trypsin/EDTA). After 3 min, 1 mL of RPMI medium was used to resuspend cells, and they were centrifuged for 5 min at 900× *g*. The supernatant was discarded, and cells were finally resuspended in 1 mL of PBS. Then, 20 μL of the cell suspension was added to 20 μL of Trypan Blue (T8154, Sigma–Aldrich, Darmstadt, Germany), and 10 μL was loaded into the cell counting slide of the TC10 cell counter (Bio-Rad, Hercules, CA, USA). Cells excluding Trypan Blue were counted as living cells.

#### 2.3.2. Flow Cytometry Analysis with Propidium Iodide

**Figure 2:**
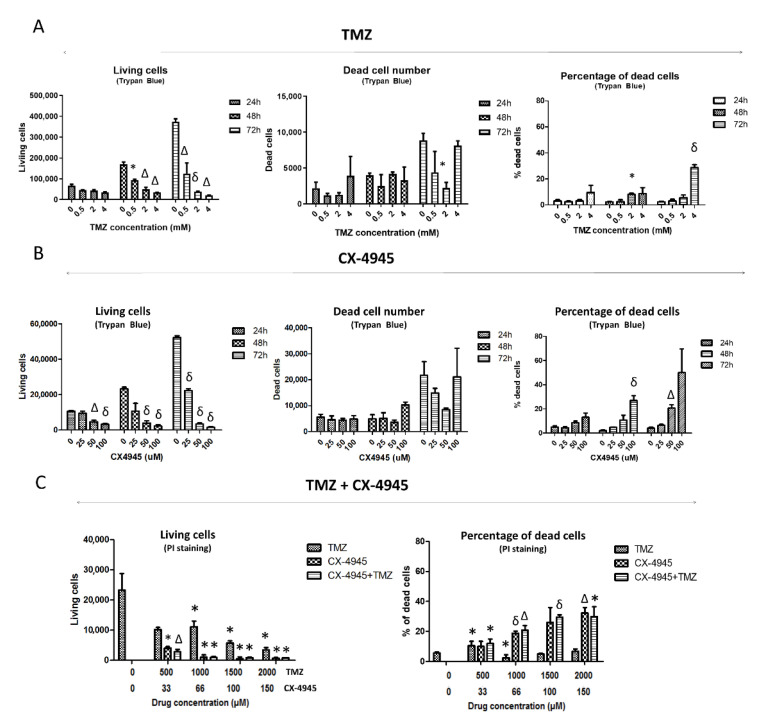
Cell viability/cell death assessment in TMZ, CX-4945, and TMZ+CX-4945-treated GL261 cells. Living and dead cell counting, as well as the percentage of dead cells assessed by Trypan Blue exclusion assay after (**A**) TMZ and (**B**) CX-4945 treatment (n = 2–3 for every case). (**C**) Living cells and propidium iodide (PI) positive cells percentage (i.e., dead cells) evaluated by flow cytometry after 72 h of TMZ, CX-4945, and TMZ+CX-4945 treatment (n = 3 for every case). Mean ± SD are shown. * (*p* < 0.05), Δ (*p* < 0.01), and δ (*p* < 0.001) mark statistical significance (Student’s *t*-test) comparing treated vs. control cells.

Propidium iodide (PI, P4170, Sigma–Aldrich, Darmstadt, Germany) was used to stain dead cells since it cannot pass through intact cellular membranes, but it is able to enter cells with damaged plasma membranes. GL261 cells were seeded in a 24-well plate at 32,000 cells per well. Cells were treated with TMZ (500–2000 µM), CX-4945 (33–150 µM), and a combination of both (see Figure 2C for further details). After treatment (12–72 h depending on the case), cells were collected, washed with PBS, centrifuged at 300× *g,* and the supernatant was discarded. Cells were resuspended in 200 μL buffer containing PBS + 2% bovine serum albumin (BSA) + PBS with 1 μg/mL of PI. Each sample was analyzed with a FACSCalibur Cytometer (Becton Dickinson, San Jose, CA, USA) for 30 s at medium speed. Values were plot as size (forward scatter, FSC) vs. granularity (SSC, side scatter) and size (FSC, forward scatter) vs. FL-2 channel intensity. Positive PI cells were considered dead, regardless of the signal intensity.

### 2.4. DAMPs Release/Exposure Assessment

DAMPs presence was assessed in GL261 cells untreated and treated with CX-4945, TMZ, or both. Drug concentrations and times of treatment will be described in detail in the corresponding subsections.

#### 2.4.1. Immunofluorescence for CRT Detection

CRT exposure in the membrane of GL261 cells was assessed with immunofluorescence. Cells were seeded in MatTek Glass Bottom Dishes (MatTek Corporation, Ashland, MA, USA). After 24h, the culture medium was replaced by medium containing treatment (TMZ 2 mM, CX-4945 25 µM and 100 µM, TMZ+CX-4945—both concentrations—and Doxo 50 µM). After 24 h of treatment, cells were washed twice with 0.5 mL of PBS and fixed for 10 min with paraformaldehyde at 2%, pH 7.4. Further on, cells were washed twice with 2 mL of ice-cold PBS and incubated with blocking buffer (2 mL of 3% BSA in PBS) for 30 min. After that, cells were incubated overnight with primary anti-calreticulin antibody from Abcam (ab2907, Abcam, Cambridge, UK) at 1:100 dilution and 4 °C and observed with a microscope Leica TCS SP5 (Leica Microsystems GmbH, Wetzlar, Germany) with a wavelength of 488 nm. The average number of cells counted was 150, depending on the treatment used in each case.

#### 2.4.2. Bioluminescent Assay for ATP Measurement

##### Extracellular ATP

For these measurements, a specific medium containing inactivated fetal bovine serum (FBS) was used to avoid ATP degradation by ATPases present in the FBS used for supplementing the culture medium. FBS inactivation was achieved by incubation for 2 h at 65 °C, as described in [38]. Cells were seeded in 24-well plates at 32,000 cells per well in a standard culture medium which was replaced after 24 h with new culture medium with inactivated FBS, and treatment was added (TMZ at 2 mM, CX-4945 at 25 µM and 100 µM, TMZ+CX-4945—both concentrations—and Doxo at 50 µM). To assess ATP degradation, the supernatant of cells growing in the medium with non-inactivated FBS was analyzed as an additional control. After the treatment period, 150 μL of cellular supernatant was collected and centrifuged at 900× *g* for 2 min to remove floating cells. Then, 100 μL of supernatant belonging to each sample was loaded in a 96-well plate, especially for luminescence assays. Victor3 Multilabel Counter (Perkin Elmer, Waltham, MA, USA) was used, and 100 μL of the ATP mix containing luciferin and luciferase was added with an automatic injection system. Immediately after addition, the generated light was measured during 10 s.

##### Intracellular ATP

For specific intracellular ATP measurement, cells were washed with PBS, trypsinized, and centrifuged. After that, the supernatant was removed, and cells were resuspended in 1 mL of Tris Borate buffer 40 mM pH 9.2 and incubated at 95 °C for 10 min for cell lysis. Further on, samples were incubated on ice for 30 s and centrifuged at 13,000× *g* at 4 °C for 5 min. The resulting cell lysate (100 μL) was used for ATP measurement as described for extracellular measurements. An estimation of the total ATP content per well was performed. As different treatments may decrease total cell number at wells, ATP content was calculated ‘per cell’. The number of living cells after each treatment was calculated from the Trypan Blue assay, and cellular water volume was estimated as ca. 1.5 pl, by comparison with C6 glioma cells volume in exponential phase [39].

### 2.5. Statistical Analysis

Sample distribution was assessed with Shapiro–Wilk or Kolmogorov–Smirnov tests, while variance homogeneity was assessed with Levene’s test. Parametric tests, such as Student’s *t*-test, were used for assessing differences in groups following normal distribution. On the other hand, for groups following non-normal distribution, nonparametric tests were performed (Mann–Whitney *U* test). For the extracellular ATP measurement, logarithm transformation of the data was used due to large variance differences among groups, as advised by our Statistical Facility (*Servei d’Estadística* UAB, http://sct.uab.cat/estadistica/, (accessed on 26 March 2021)). After transformation, the Kolmogorov–Smirnov test and Levene’s test were used for assessing sample distribution and variance homogeneity, respectively. The analysis of variance (ANOVA) test was applied with Tukey’s post-hoc correction for multiple comparisons.

## 3. Results

In this work, the immunogenic signals triggered by TMZ, CX-4945, and the combination of both drugs were studied in GL261 cells in vitro. First, the effects of these treatments on the GL261 cell viability were assessed.

### 3.1. Effects over GL261 Cell Viability

#### 3.1.1. TMZ Single Treatment

To characterize the TMZ effect on GL261 cells and to elucidate whether a cytostatic or cytotoxic effect was taking place in those cells, treated cells were stained with Trypan Blue and analyzed by manual cell counting after 24, 48, and 72 h of treatment at 0.5, 2, and 4 mM (Figure 2A). The living cell counting decreased significantly after 48 and 72 h of treatment at every concentration studied. No significant changes were found in dead cell counting, except at 2 mM when a significantly lower number of dead cells was observed when compared to control cells. This apparent inconsistency may be caused by spontaneous cell death taking place in control cells since their total cell counting is clearly higher than treated cells. Thus, the total number of dead cells is not suitable to elucidate whether a drug is producing a cytotoxic effect. On the other hand, the percentage of the dead cells may be more useful for this purpose since it relates the dead cell counting to the total cell number. In this respect, no increase in cell death percentage was seen for 0.5 mM TMZ at any incubation time. Still, such percentage increased from 3 to 8% after 48 h at 2 and 4 mM, respectively. After 72 h, the dead cell percentage at 4 mM was 27%, thus indicating a possible cytotoxic effect of TMZ when used at 4 mM. However, we should also consider that the concentration described to have been reached following in vivo administration of TMZ at standard doses of 60 mg/Kg for mice (250 µM, according to [40], also in agreement with values described in [41]), is much lower than 4 mM, and below the 0.5 mM value at which no increase in cell death was detected.

Propidium iodide (PI) staining was also used in TMZ-treated cells, which were analyzed with flow cytometry after 72 h of treatment at 0.5, 1, 1.5, and 2 mM (Figure 2C). A decrease in living cell counting was observed at 1, 1.5, and 2 mM, while the percentage of dead cells increased from 6 up to 10% at 0.5 mM. Although this was statistically significant, in our opinion, it is not enough to identify TMZ as a cytotoxic agent for in vivo work because most cells remained alive, no significant cell death increase could be monitored for higher TMZ concentrations (1–2 mM) with respect to control values, and TMZ exposure in vivo is not continuous for 72 h (see also Section 4.4). In short, these results taken together suggest that TMZ could have a cytostatic rather than a cytotoxic effect in GL261 cells evaluated in vitro at the concentrations achieved during in vivo administration (ca. 250 μM [40]). This was further confirmed in other in vitro studies from our group (not shown) in which GL261 cell treatment with TMZ 298 μM during 72 h had only minor effects over cell viability (88% cell viability found with Trypan Blue).

#### 3.1.2. CX-4945 Single Treatment

GL261 cells treated with CX-4945 were collected, and a Trypan Blue exclusion assay was performed (Figure 2B). The living cell overall counting decreased significantly after 24 h of treatment, being higher at longer treatment times. No differences were found for dead cell counting between untreated and CX-4945 treated cells. With respect to the percentage of Trypan Blue positive dead cells, it was found to be significantly higher after 48 h of treatment and at the highest concentration analyzed (100 μM), with an average value of 27% of dead cells. After 72 h of treatment, the dead cell percentage was 21% at 50 μM and 50% at 100 μM, although significance was not reached in the latter in comparison to untreated cells due to high data values’ dispersion.

Results showed that CX-4945 mainly produced a cytostatic effect reducing the number of living cells, especially at low concentrations. On the other hand, a higher cytotoxic effect than with TMZ (about 50% dead cells) was observed with the use of high concentrations and longer treatment times (Figure 2B).

#### 3.1.3. CX-4945 and TMZ Combined Treatment

In this work, the cytotoxic potential of the single and combined treatments was assessed through flow cytometry with PI-stained cells after treatment with TMZ, CX-4945, and a combination of both. The living cell counting was similar when comparing cells treated with CX-4945 and the combination TMZ+CX-4945 (the maximum difference found being 5.2%, non-significant). The dead cell percentage was 26.6% ± 9.9 for 100 µM CX-4945 and 29.6% ± 1.6 for the combination (same concentration of CX-4945 plus 1.5 mM TMZ), suggesting a discrete, although non-significant, synergistic effect between them, showing a coincident trend with [19]. Results suggest that the combined treatment has a slightly more pronounced effect, albeit non-significant, than the single CX-4945 treatment in cell viability, both being more cytotoxic than TMZ alone (Figure 2C).

### 3.2. Immunogenic Cell Death Signals Triggered In Vitro under TMZ and CX-4945 Treatment Alone and in Combination

We assessed the ability of CX-4945, TMZ, and combined TMZ+CX-4945 in vitro treatments to produce immunogenic cell death and lead to the exposure/release of immunogenic signals. This could help to explain the beneficial results obtained with IMS therapeutic schedule in GL261 tumor-bearing mice and to provide a better understanding of the combined effects in vivo already described by us [29].

#### 3.2.1. Calreticulin Exposure

**Figure 3:**
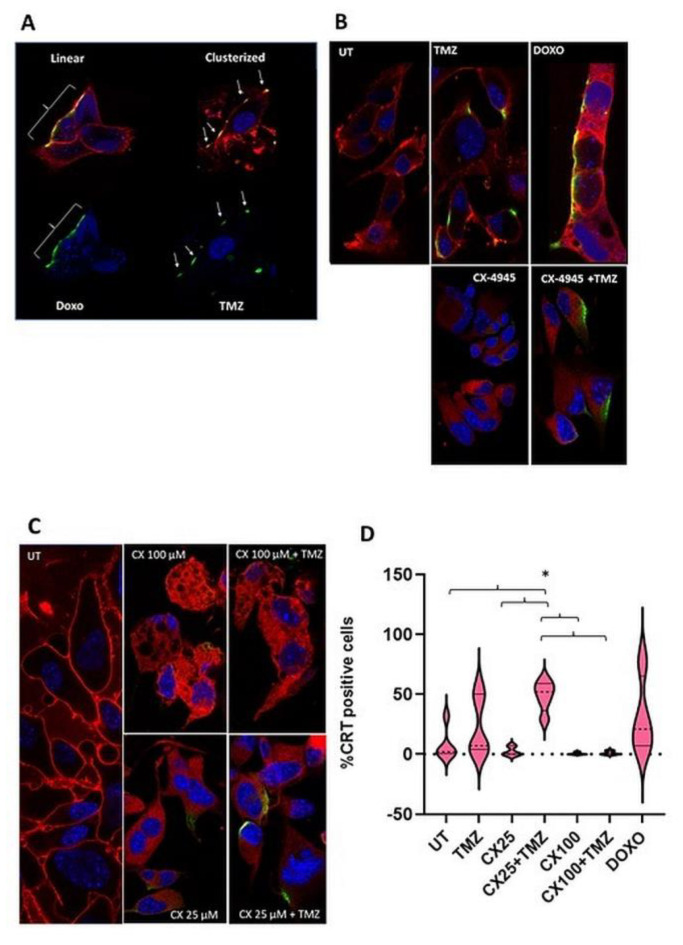
CRT exposure in the GL261 cells plasma membrane after treatment. Nuclei were stained with Hoechst dye 33,342. White brackets and arrows indicate lineal or clusterized CRT, respectively. Membranes were stained with Cell Mask and shown in red. (**A**) Example of CRT exposure in its different conformations. Top: membranes are shown in red, nuclei in blue, and CRT in green, with the overlay CRT/membrane shown in yellow. Bottom: only nuclei (blue) and CRT in green are shown. On the left side, Doxo-treated cells (50 µM, 24 h of treatment) show CRT in a linear, elongated conformation (green). On the right side, TMZ-treated cells (2 mM, 24 h of treatment) show a clusterized, focal pattern of CRT exposure. TMZ-treated cells had nuclei stained with Hoechst dye. In Doxo-treated cells, Doxo prevents the union of Hoechst and the nuclear structures. Hence, blue staining corresponds to autofluorescence of the nuclei-bound Doxo itself. (**B**) Immunofluorescence staining for all conditions studied. All treatments were performed for 24 h: TMZ (2 mM), CX-4945 (CX, 25 µM), TMZ+CX-4945 (2mM, 25 µM) and Doxo (50 µM). (**C**) Comparison between CRT exposure staining in 24 h treatments of CX-4945 and CX-4945+TMZ at two different concentrations of CX-4945 (CX, 25 and 100 µM). (**D**) Violin plots indicating the quantitation of CRT-exposing GL261 cells upon treatment with the conditions explained in (**B**,**C**). The percentage of positive cells is shown (n = 3–5 for each case). Median is shown in dotted lines, while quartiles are shown with continuous lines. UT, untreated cells. “*” states for *p* < 0.05 for the indicated comparisons.

CRT exposure is an initial event in the ICD cascade acting as a potent “eat-me” signal [42]. This exposure has been analyzed by confocal microscopy in GL261 cells, both untreated and treated with TMZ (2 mM), CX-4945 (25 and 100 µM), and CX-4945+TMZ (all combinations) after 24 h of treatment. Cells treated with doxorubicin (Doxo) were used as a positive control since they have been described to trigger CRT exposure [43,44]. This can be seen with GL261 cells in Figure 3A (white brackets and arrows). At the assayed Doxo concentrations, most (75–90%) of GL261 cells evaluated by us should be dead/dying, and concentrations were far above the described EC50 [44].

For estimation and counting of confocal fluorescence microscopy observations, cells with an absent CRT signal in their membrane were considered negative (i.e., no exposure) (e.g., Figure 3B, most of untreated (UT) cells). It is worth noting that CRT exposed cells in Doxo and TMZ-treated samples presented large value dispersion, as can be seen in the violin plots (Figure 3D), and the registered values did not follow a normal distribution. Accordingly, nonparametric tests were used to compare untreated cells with the other groups. Untreated cells showed low percentages of CRT exposed cells (7.9 ± 13.5% on average), which was considered as the GL261 cell line basal CRT exposure. Doxo-treated cells presented a different behavior: higher percentages of CRT-exposing cells were observed (average ca. 4-fold increase, 30.6 ± 32.4% of cells), although non-significant due to the dispersion of values. In this sense, Doxo-treated cells were considered a suitable positive control for the protocol used in this work.

Regarding TMZ-treated cells, CRT positive cells were present in a higher percentage as compared to UT cells (ca. 3-fold change, 23.0 ± 25.0%). As for Doxo treated cells, due to the large data dispersion, no significant differences were found. Moreover, CX-4945 treated cells were also analyzed at two different concentrations, 25 µM (Figure 3B) and 100 µM (Figure 3C). At both concentrations, CX-4945 single treatment triggered CRT exposure in a small number of treated cells, with average values even lower than untreated cells, albeit not significantly different. The combined CX-4945+TMZ treatment (with CX-4945 at 25 µM) increased the percentage of CRT-exposing cells significantly when compared with single treatments (20-fold increase with respect to CX-4945 alone and ca. 2-fold with respect to TMZ alone). Regarding the combined treatment with the higher concentration of CX-4945 (100 µM), CRT exposure was not significantly different from the UT or CX-4945 treatment alone at this concentration (Figure 3C,D).

#### 3.2.2. Assessment of ATP Release

Released ATP has been described as stimulating the immune response acting as a short-range “find-me” signal with a proinflammatory role by binding to P2Y2, P2X7, and P2 receptors [45] found in dendritic cells (DCs) and monocytes.

The content of ATP in the supernatant of the GL261 cells was measured with a luciferin–luciferase based assay in TMZ, CX-4945, and TMZ+CX-4945 treated cells. Since TMZ needs more time to decrease cellular viability (Figure 2), we treated cells with TMZ for 24 h and, after this period, CX-4945 was added. Three different time points were studied: 12, 18, and 24 h after CX-4945 treatment. The TMZ concentration used was 2 mM, and CX-4945 was administered at two different concentrations, 25 and 100 µM, to be consistent with CRT exposure studied concentrations.

**Figure 4:**
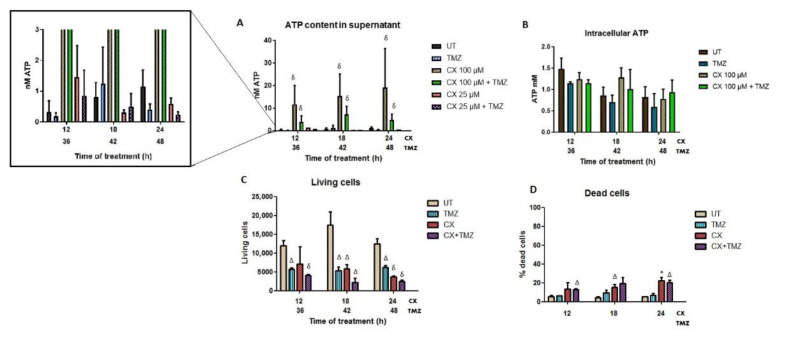
ATP quantification in GL261 treated cells (supernatant release and intracellular content), as well as cell viability assessment after treatment. CX-4945 (CX, 100 and 25 µM), TMZ (2 mM), and combined CX-4945+TMZ treatments were evaluated. (**A**) ATP content released in the cell culture supernatant. Average ± SD values are shown for n = 3–6 in every condition. δ indicates *p* < 0.001 according to ANOVA (Tukey’s post-hoc comparisons) vs. TMZ and untreated cells. No significant differences were found between single CX-4945 and joint CX-4945+TMZ treatments. An expansion is shown on the left to appreciate values with small values better (higher values will be out of scale) (**B**) Content of intracellular ATP in GL261 treated cells after treatment, (n = 3). Average ± SD is shown, with no significant differences found. (**C**) Living cells after CX (100 µM), TMZ (2 mM), and combined treatment CX+TMZ (100 µM and 2 mM), analyzed by flow cytometry after PI staining, (n = 3 for each condition). (**D**) Percentage of dead cells after treatments. Average ± SD is shown for all cases. *, Δ and δ indicate significance (*p* < 0.05, *p* < 0.01 and *p* < 0.001, respectively) according to Student’s *t*-test, for comparisons between treated and untreated cells.

Results showed that both CX-4945 and CX-4945+TMZ treated cells, at the highest concentration of CX-4945 (100 µM), released significantly more ATP compared to untreated or TMZ-treated cells (Figure 4A) for every time point studied. However, the maximum difference in ATP release when compared to untreated cells was found after 12 h of treatment (ATP content was found to be 37-fold higher for single CX-4945 treatment and ca 12-fold higher for the combined treatment). Moreover, single CX-4945 treatment was significantly different from combined CX-4945+TMZ at some time points, as follows: for the 100 µM concentration, differences were either significant (*p* < 0.05, 12 h) or tending to significance (0.05 < *p* ≤ 0.1, 18 and 24 h). For the 25 µM concentration, only the 24 h time point showed significance. The lower ATP release values observed after the combined treatment indicate that the combination of CX-4945 with TMZ triggered a 3-fold decrease in the overall amount of ATP released, although still significantly higher than in untreated cells.

However, no differences were found at the lower concentration of CX-4945 (25 µM) in comparison with UT cells, indicating that this concentration was not enough to trigger ATP release in the concentrations and time points assayed. On the other hand, single TMZ treatment somewhat decreased the amount of ATP released (0.32 nM for UT vs. 0.17 nM for TMZ, non-significant, *p* > 0.3) at every time point studied. One possible reason could be the production of a different immunogenic signal related to ATP consumption or some interference with the ATP release in TMZ-treated GL261 cells. In addition, intracellular ATP and the estimated amount of ATP per cell were also measured (Figure 4B). Only the highest concentration of CX-4945 was evaluated since it was the only concentration that triggered significant ATP release. No significant differences were found among the different treatments, with no noticeable changes in the intracellular ATP content, as opposed to the extracellular released ATP.

##### Cytotoxicity Assessment after Short Treatment Times: Can It Affect ATP Release?

ATP is presumably released either actively by pre/early apoptotic cells [46] or through passive release in necrotic cells [47]. One of the questions raised in this work was whether the ATP release measured after treatments originated in living or membrane-compromised cells. To elucidate this, CX-4945, TMZ, and CX-4945+TMZ treated cells were analyzed by flow cytometry after incubation with PI in the same conditions, and results are shown in Figure 4C,D. CX-4945 treated cells were only analyzed at the highest concentration (100 µM) since it was the only one triggering significant ATP release.

The maximum amount of ATP released was observed after 24 h of CX-4945 treatment in comparison with untreated cells. Flow cytometry results after CX-4945 and CX-4945+TMZ treatments showed 22.3% and 20.5% of dead cells, respectively, indicating that the remaining cells were still alive after 24 h. Thus, assuming the contribution of dying cells to extracellular ATP is similar in the two treatments, the larger ATP content in the supernatant must originate mostly from living cells (see also discussion in this regard).

## 4. Discussion

### 4.1. TMZ Is Not Enough: Pursuing Better Combinations/Schedules

Despite the significant efforts made to improve therapeutic approaches and schedules for GB, its prognosis is still very poor in the clinical scenario. TMZ has been widely studied both in vitro and in vivo for GB treatment. Its beneficial effect is, in most studies, attributed to its effect as an alkylating agent able to activate the DNA repair mechanisms of the cells and, subsequently, the apoptotic cascade [4,48]. From our results, it is clear that TMZ alone, given to GL261 cultured cells in vitro, at concentrations similar to concentrations reaching the tumor when used in preclinical studies in vivo, has a cytostatic rather than a cytotoxic mechanism, in agreement with work by others [9,49,50]. The local concentration needed to produce some degree of cell death, about 2 mM, is definitely beyond the concentrations achieved in either preclinical or clinical settings (e.g., [9,51]). Thus, it is clear that a different mechanism is needed to explain the in vivo effects of TMZ, such as ICD/DAMP release. However, the potential and promising effects of TMZ as an immunogenic drug have not attracted much attention, although this is gaining prominence nowadays [9]. Moreover, although TMZ is currently the best chemotherapeutic option approved for GB, its beneficial effect is clearly insufficient. For instance, the median overall survival for GB with TMZ plus radiotherapy treatment did not really improve in terms of years (14–17 months) [52,53]. For this reason, combination schedules with drugs showing different mechanisms of action have been explored in preclinical models, such as the joint treatment with small molecular weight CK2 inhibitors [23,29] or the use of immune checkpoint inhibitors such as anti-PD1 [9,54,55,56].

### 4.2. Immunogenic Potential of Single and Combined Treatments and Possible Pitfalls While Interpreting Results

In this work, we showed that both TMZ and, especially, joint TMZ+CX-4945 treatment of GL261 cells trigger CRT exposure on their surface. This CRT may act as a potent immunogenic “eat-me” signal for APCs and could be a key element in recruiting the immune system to produce the TMZ-related outcome in GB. In addition, having in mind that our results indicate that, at least in vitro, TMZ at the concentrations circulating in living mice models (about 0.3 mM [40,41]) mostly acts as a cytostatic agent, we need a different source of cytotoxicity to explain its therapeutic effect. Thus, in agreement with results in [9], it would be mostly TMZ-induced signaling recruited immune system cells (CTILs and macrophages), being the ones killing GL261 cells directly during the response to therapy, rather than TMZ direct activity over tumor cells.

Furthermore, GL261 cells treated with CX-4945 (alone or in TMZ combination) are shown here to induce ATP release from living GL261 cells. This is a strong “find-me” DAMP that provides a chemotactic guide in the tumor surrounding tissue and within the tumor to facilitate APCs reaching the vicinity of damaged tumor cells exposing CRT “eat-me” signals. Thus, both signals, exposed CRT and released ATP, support the proposed role of TMZ and CX-4946 agents in attracting immune system cells to the vicinity of damaged/dying tumor cells to facilitate adequate priming and amplification of lymphocytes within the cancer immune cycle summarized in Figure 1.

One could also hypothesize whether the increase in ATP content in the supernatant of cultured GL261 cells could be simply a consequence of the increased amount of dead cells due to different treatments. In this respect, apart from the considerations mentioned in Section 3.2.2, it is well known that under a certain ATP threshold (ca 15% of the basal concentration [57]), cells may undergo necrosis, which in the end would release the remaining ATP content from the cell. Thus, taking as an example the comparison between UT cells and CX-4945 100 µM treatment, overall dead cell count only increased ca 1.4 fold, while the extracellular ATP content increased 37 fold. Hence, although a small part of the measured extracellular ATP can be indeed contributed by necrotic dying cells, most of the detected ATP in our conditions must have arisen from an active release from living cells. Several cellular mechanisms that could explain this release have been explained in detail in [58], and in this respect, it worth mentioning that the amounts of ATP measured were far below the threshold in which it could instead have immunosuppressive effects [59]. Additionally, the 12 h timing high mark agrees with the one reported for ATP release in C6 rat glioma cells after a targeted liposomal treatment [60]. Moreover, ATP released by glioma cells has been described to help in microglia/macrophage activation and production of macrophage inflammatory proteins [61].

**Figure 5:**
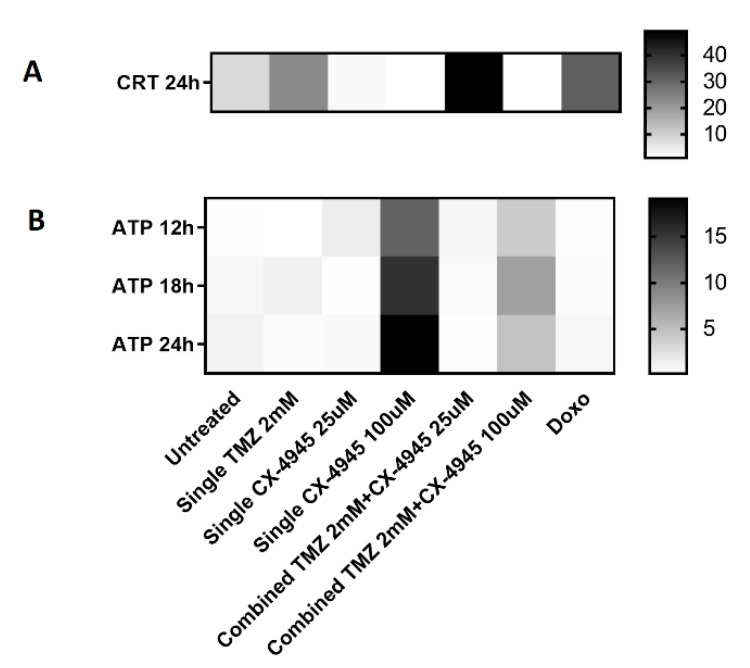
Heatmap for comparison of (**A**) CRT exposure and (**B**) ATP extracellular release in the different conditions studied. CRT is expressed in the same units as in Figure 3D, and ATP release in the same units as in Figure 4A. Hence, they are shown using different scales (columns at right). For Figure 5B, TMZ was also added 24 h before CX-4945, and the overall time passed is thus different for TMZ and CX-4945, as already stated in the Figure 4 legend.

Furthermore, it is worth noting that, as summarized in Figure 5, the release of immunogenic signals by GL261 cells was both time and concentration-dependent. This is consistent with data from other authors. Thus, TMZ triggering CRT exposure in GL26 mouse GB cultured cells was already described by Kim et al. [6], although using longer treatment times than in our case (72 h). Others have gone a step beyond and proved that CRT exposure due to TMZ was observed in different GB cell lines, associated with endoplasmic reticulum stress [9]. In their case, the point of the maximum percentage of CRT-positive cells varied with the cell line (48–72 h) with average values similar to the ones described in this work. TMZ was also shown by them able to promote GB cell phagocytosis by bone marrow-derived phagocytes, reinforcing the key role of the innate immune system in response to TMZ therapy [9]. The CRT exposed in the plasma membrane was described as assembling in clusters hence facilitating the binding to the APCs receptor CD91 [62], triggering subsequent presentation of tumor-associated antigens to T lymphocytes [63]. First, CRT would be transported through the ER-Golgi pathway [46] towards the plasma membrane, first being organized in a linear shape and then further clusterized. This type of organization can be seen in Figure 3B (for TMZ treatment). Such CRT exposure was absent in our hands with CX-4945 treatment alone, but it was maximal in the combined TMX/CX-4945 treatment (Figure 3B,D). This indicates that the joint treatment allows better CRT exposure, could contribute, at least partially, to improved immune system recruitment into tumors, and explain a better survival of mice under the joint treatment compared to single IMS TMZ in vivo [29].

Regarding ATP release, and considering the discouraging results obtained with in vivo CX-4945 treatment of GL261 GB tumor-bearing mice [29], it seems clear that released ATP alone cannot be the major determinant immunogenic signal involved in GL261 GB treatment, needing the participation of other DAMPs for an improved outcome.

### 4.3. Word of Caution While Interpreting the Additive Effect of Immunogenic Signals In Vitro

In this work, we have shown that treatment of GL261 cells with 100 µM CX-4945 triggered significantly more ATP release when compared to untreated cells or at 25 µM, either alone or in combination with TMZ. On the other hand, regarding CRT exposure, cells treated with TMZ plus the lowest concentration of CX-4945 (25 µM) did show optimal CRT exposure while combining TMZ with high CX-4945 (100 µM) did not expose CRT. Thus, we seem to need high CX-4945 (100 µM) to induce high ATP “find me” signal optimal release, 25 µM CX-4945 is not enough for that. The added TMZ seems to partially inhibit ATP release induced by 100 µM CX-4945. On the other hand, combined TMZ and low 25 µM CX-4945 was optimal for CRT exposure, while combined TMZ and high 100 µM CX-4945 fully inhibited CRT induced exposure over untreated GL261 cells. So, treatment conditions which seem optimal for “find me” immunogenic signaling displayed through ATP release (100 µM CX-4945 alone or with TMZ) will make the “eat me” CRT signaling disappear, while conditions optimal for “eat me” signaling by CRT exposure (25 µM CX-4945 with TMZ) will lose the “find me” signaling through released ATP. This apparent discrepancy between in vivo CX-4945 and TMZ synergy [29] and in vitro results can have different explanations. In the first place, the concentrations of TMZ and CX-4945 reaching GL261 tumors in vivo and their pharmacokinetics in [26] are not known. Accordingly, the concentrations used in our in vitro experiments may not adequately mimic the in vivo situation (see also Section 4.3).

In the second place, different sensitivity intrinsic to the detection methods used can hamper the adequate interpretation of some of the results described in this study. Thus, ATP measurement by luminescence may be less sensitive than the evaluation of CRT exposure by confocal microscopy. Moreover, confocal microscopy allowed studying individual glioma cells, while in the ATP released measurements, a pool of cells was being studied, and some heterogeneity can take place (i.e., some cells releasing ATP and others not, making the overall result an average). It is known that the combined action of endoplasmic reticulum (ER) stress and reactive oxygen species (ROS) production is a prerequisite for the exposure and release of DAMPs to happen [42]. In this respect, Intemann et al. [64] described that CX-4945 treatment in several cell lines triggered ER stress, which would reinforce CX-4945 as an immunogenic drug in GB therapy, although, in our hands, it did not induce CRT exposure when used alone.

A heatmap summarizing the main immunogenic signals observed in the different conditions studied is shown in Figure 5. The fact that the joint treatment did not prevent the appearance of both immunogenic signals (see also Section 4.3 for comments about the actual concentration of both agents in in vivo experiments) could possibly explain the reason for the synergistic action described in [29].

Acknowledgment of immune system participation in cancer therapy response is nowadays an accepted issue [65,66,67]. For proper immune system elicitation, the correct appearance of immunogenic signals should be triggered, and, in addition, no antiproliferative treatment during the amplification stage of the cancer immune cycle (Figure 1C) must be performed, for example, using the IMS schedule [35].

### 4.4. In Vitro Studies Do Not Properly Mimic the In Vivo Environment

In previous work provided by us [29], the combined in vitro CX-4945+TMZ treatment showed better results than single treatments, using MTT assay approaches. Namely, the combined treatment proved to decrease 4.5 fold cell viability in comparison with untreated cells, whereas treatment with CX-4945 alone caused a 3-fold decrease, and TMZ alone caused only 1.7 fold-decrease.

We were interested in elucidating the effect of TMZ in C57BL/6 mice harboring GL261 GB since this is the standard, and so far, best, chemotherapeutic treatment for human GB. However, it is worth noting that the preclinical model treatment did not fully replicate the therapeutic scheme performed in clinical settings. In human GB, TMZ is usually administered after tumor resection and/or in combination with radiotherapy, conditions that are not usually fully simulated, neither in GL261 GB nor, of course, in in vitro studies. Moreover, it is relevant to have in mind that in vitro studies hardly reproduce actual in vivo conditions, even preclinical ones. During in vivo therapy, drugs are administered and absorbed with different rates and half-lives, exposing the cells to determined temporary concentrations followed by washout, while in vitro studies provide constant concentrations of therapeutic compounds.

If we compare CX-4945 hypothetical in vivo concentrations expected to be reached after oral administration (such as the one used in [29]) and also taking into account the pharmacokinetic profile [68], the CX-4945 plasma concentration in mice would have been 6.16 mM, far beyond concentrations assayed in our experiments (25 and 100 µM). Even though the hypothetical concentration in plasma of CX-4945 would be very high according to such previous literature, it is important to take into account that these authors also found out that almost all CX-4945 (98.8 ± 1%) was in a bound form in the plasma, which could finally prevent most drug reaching the tumor site. Nevertheless, even if only a small fraction of the administered drug reached the tumor, we could still hypothesize that tumor concentration would probably be a cytotoxic one for CX-4945 (i.e., >100 µM). The opposite should take place for TMZ. Concentration values expected after an in vivo administration (250 µM, according to [40], also in agreement with [41]) would be somewhat lower than the lowest concentrations studied in vitro in this work.

Additional factors may affect drug bioavailability. For instance, drugs should face heterogeneous environments, with pH gradients and the presence of normal, non-tumoral cells and cells from the immune system infiltrating the tumor and competing for the drug. Thus, we can probably assume that the immunogenic signals detected in in vitro work will also be produced in vivo, but, having in mind different timing related to drugs half-life and bioavailability, this may mean that the drug and the induced DAMPs are not necessarily going to be present at the same time in the tumor environment. Finally, the introduction of different environmental factors, such as the “enriched environment” for animal housing [69], described to improve the performance of immune system cells, has had a clear impact on mice survival upon treatment [35], but it is hardly reproduced in in vitro work.

## 5. Conclusions

In conclusion, the in vitro study performed with cultured GL261 GB cells indicated that TMZ treatment is able to trigger CRT exposure, while CX-4945 produces ATP release. A combined treatment using the two drugs may trigger both ICD signals, being a possible explanation for the beneficial effects detected in preclinical GL261 GB survival [29]. The effect of TMZ at the concentrations assumed to reach the tumor in vivo, would be essentially cytostatic, but immunogenic through CRT exposure, attracting APCs and macrophages. On the other hand, ATP release following CX-4945 treatment could reinforce the attraction of APCs to the CRT exposure site. To the best of our knowledge, this is the first study reporting production of classical immunogenic signals upon CX-4945 administration, although probably ATP alone is not enough to elicit a potent immune system response since only modest survival increases were obtained even with an IMS administration [26]. Translational studies that do not take into account the required synchronicity with the cancer immune cycle could fail, discarding an a priori promising drug that could present satisfactory results provided the correct timing in therapeutic schedules is respected. Finally, the mounting of suitable host immune response against GB tumors in immunocompetent models may benefit from combined TMZ and CX-4945 use.

## Figures and Tables

**Table 1:** Drug concentrations and treatment time ranges used in this study.

Drug	Range of Concentrations Assayed	Range of Treatment Time (h)
TMZ	0.5–4 mM	24–72
CX-4945	25–150 µM	12–72
Doxorubicin	50 µM	24

## Data Availability

Data will be made available through UAB Digital repository (DDD UAB https://ddd.uab.cat/, (accessed on 25 March 2021)).
